# Economic evaluation of the ‘Click & Crunch’ online intervention to improve healthy food purchases from Australian primary school canteens: a cost and cost-effectiveness analysis

**DOI:** 10.1017/S1368980026102286

**Published:** 2026-03-25

**Authors:** Rebecca Wyse, Tara Clinton-McHarg, Rachel Zoetemeyer, Rachel Sutherland, Kathryn Reilly, Tessa Delaney, Serene (Sze Lin) Yoong, Luke Wolfenden, John Wiggers, Penny Reeves

**Affiliations:** 1 School of Medicine and Public Health, The University of Newcastle, Callaghan, Australia; 2 Hunter New England Population Health, Wallsend, Australia; 3 Hunter Medical Research Institute, Newcastle, Australia; 4 Melbourne School of Population and Global Health, The University of Melbourne, Parkville, Australia; 5 Global Centre for Preventive Health and Nutrition, Deakin University Faculty of Health, Geelong, Australia

**Keywords:** Cost-effectiveness, Costing, Healthy eating, Online ordering systems, Nudge, Choice architecture, Behaviour

## Abstract

**Objective:**

Behavioural interventions can be delivered via online school canteens to improve healthy purchasing for students. However, no evaluations of the cost or cost-effectiveness of online canteen interventions have been conducted. The objective was to conduct a cost and cost-effectiveness analysis of implementing an online school canteen intervention to improve healthy purchasing.

**Setting:**

The ‘Click & Crunch’ cluster randomised controlled trial was conducted in seventeen Australian primary schools.

**Participants:**

Eight control schools (848 students) received the standard online canteen. Nine intervention schools (1359 students) received a behavioural intervention delivered through the online canteen.

**Design:**

Incremental cost-effectiveness ratios (ICER) were calculated for the cost per student to achieve (1) a unit decrease in the mean energy (kJ) content and (2) a percentage increase in the mean proportion of healthier ‘Everyday’ items purchased in their online lunch orders (from a health service and societal perspective).

**Results:**

It costs AUD$568 per school (range $343–$806) to implement. The ICER for mean energy content was AUD$0·06 and AUD$0·46 for mean proportion of ‘Everyday’ foods (from a health perspective). At a willingness to pay of AUD$0·20 and AUD$1·20 per student, the intervention would have a 95 % and 99 % probability of being cost-effective for the energy (kJ) content and proportion of ‘Everyday’ items, respectively.

**Conclusions:**

‘Click & Crunch’ has the potential to be a cost-effective intervention to reduce the energy content and increase the proportion of ‘Everyday’ items from primary school online canteen lunch orders.

Dietary risk factors are a leading preventable cause of disease internationally, contributing to over 11 million deaths and 255 million disability-adjusted life years^(1)^, predominantly due to chronic disease and obesity-related conditions^(2)^. This burden comes at a tremendous expense, with the cost of disease and death due to diet- and obesity-related issues estimated to be USD $311 billion annually (cost-year 2019) in Organisation for Economic Cooperation and Development countries^(3)^. Dietary habits are formed in childhood and track into adulthood, and childhood dietary patterns are associated with chronic disease in adults^(4,5)^. The majority of children in developed countries, including Australia, fail to meet dietary guidelines for core foods such as fruit and vegetables^(6,7)^ and consume excess saturated fat, sugar and salt^(6,8,9)^. As such, interventions in childhood are needed to improve public health nutrition and protect against the onset of preventable diet-related chronic diseases^(2)^.

Technology-based dietary interventions are particularly appealing given their acceptability, accessibility and scalability^(10)^. While the use of technology to deliver dietary interventions is not new (e.g. websites, gamification, apps to provide training, monitoring, peer support), a more recent development is the use of technology to select and order food, for example through online menu ordering systems^(11)^. The number of users of such systems has grown rapidly in recent years^(12)^, and this growth is predicted to continue^(13)^. This provides a novel opportunity for dietary interventions to be embedded into existing online systems with an established, sizable and regular user base^(14)^. Although it has been suggested that digital interventions^(15,16)^, and particularly those embedded into existing online ordering systems^(14)^, can achieve widespread health behaviour change at a relatively low cost, there is little empirical evidence to support this^(17,18)^. A recent review of choice architecture interventions embedded within food ordering systems found that no economic analyses had been conducted for any of the eleven included studies^(11)^.

Traditionally, schools have been an important setting in which to implement dietary interventions targeting children^(19)^. Schools provide access to almost all children, for large periods of time, and are a key setting for dietary intake^(20)^. While some countries have school food programmes that provide meals to all children attending school^(21)^, other countries, including Australia, have a school canteen or kiosks where students can purchase food and drink instead of, or in addition to, bringing meals from home^(22,23)^. Increasingly, school canteens are offering online ordering, which enables users (e.g. parents/carers and/or children) to pre-order and pay for food and drink that students can access and consume later during school break times^(24)^. The reach of online canteens is growing, with the leading provider of online canteens in Australia, supporting approximately 1400 schools nationally and processing approximately 13 million orders for 400 000 users annually^(25)^.

Given the prevalence of dietary risk factors in childhood, it is vital that effective dietary interventions have the capacity to be enacted on a large scale and represent judicious use of scarce health and school resources. Cost-effectiveness analysis estimates and compares the costs and health gains of interventions, thus helping to identify those with the potential to yield the highest health gains using the least resources, and is recommended to inform health care decisions^(26)^. However, to the authors’ knowledge, there is no published literature investigating the cost and cost-effectiveness of implementing school-based dietary interventions using online canteens. A systematic review of 23 economic evaluations of school-based lifestyle interventions among 4–12 years olds^(27)^ included only two studies which included an online strategy^(28,29)^ and only as part of multi-component interventions. No studies of school-based online lunch-ordering interventions were included in the review. A systematic review of 137 behavioural insights interventions (i.e. subtle modifications to the social and physical environment to prompt behaviour change) to influence child diet identified only seven studies that reported implementation cost or cost-effectiveness^(30)^. However, none of these interventions were delivered online. Furthermore, a number of systematic reviews have been conducted to determine the effectiveness of digital dietary and obesity interventions for children^(10,31–35);^ however, none of these included any lunch-ordering interventions, and no studies of cost-effectiveness were included.

In view of the limited evidence regarding the cost-effectiveness of online dietary interventions in schools, an economic evaluation of an online intervention to improve healthy food purchases from primary school canteens was conducted. In view of the limited evidence regarding the cost-effectiveness of online dietary interventions in schools, an economic evaluation of an online intervention to improve healthy food purchases from primary school canteens was conducted. ‘Click & Crunch’ was a healthy eating intervention that involved embedding choice architecture strategies (i.e. menu labelling, item positioning, prompting, feedback, incentives) in an online school canteen ordering system. It was evaluated in a trial involving seventeen Australian primary schools (see ‘Click & Crunch Intervention’ below). Specifically, the aims of this economic evaluation are to determine the cost and cost-effectiveness, from a health and societal perspective, of the ‘Click & Crunch’ online canteen intervention in terms of 1) reducing the mean energy (kJ) content of primary school students’ online canteen lunch orders and 2) increasing the average proportion of healthier ‘Everyday’ items within the online lunch order, relative to standard online canteen lunch orders.

## Methods

### Study design and setting

A trial-based economic evaluation was undertaken from a health service and societal perspective (which includes costs to both the health service and to the school) to estimate the cost of implementing the ‘Click & Crunch’ online canteen intervention compared to usual practice (the standard online school canteen ordering system) in seventeen primary schools in New South Wales, Australia. Details of the planned economic evaluation were included in the prospective trial registration (ACTRN12618000855224). The study was approved by the Human Research Ethics Committee of The University of Newcastle (H-2017–0402) and the Ethics Committees of the relevant Catholic Education Office Dioceses. The study was reported against the Consolidated Standards of Reporting Trials^(36)^ guidelines and the Consolidated Health Economic Evaluation Reporting Standards^(37)^ statement.

### Intervention trial

#### Participants and recruitment

Non-government (Catholic or Independent) schools were eligible to take part in the study if they enrolled primary school students from Kindergarten to Grade 6 and used an online canteen ordering system. ‘Combined’ schools, enrolling both primary and high school students, were eligible provided they had separate canteen menus for primary and high school students (due to differences in the state-wide New South Wales (NSW) Healthy School Canteen Strategy for primary and high schools). Potentially eligible schools were invited to participate by mail and telephone.

Student orders were included in the analysis if they had been made via a mobile phone app, were therefore exposed to all intervention strategies (around 70 % of all orders are made this way), were placed for a lunch break on a usual canteen day (e.g. not a special food day), and contained a plausible number of items (i.e. fifteen items or less). Fifteen items were set as the cut-off as some canteen items are purchased as individual units (e.g. six chicken nuggets).

#### Click & Crunch intervention

‘Click & Crunch’ is an effective multi-strategy behavioural intervention embedded within an established online canteen ordering system, that aimed to decrease the energy, saturated fat, sugar and sodium content of online lunch orders for primary school children and to increase the proportion of healthier ‘Everyday’ foods purchased while reducing the proportion of less healthy foods purchased. The trial methods are fully described in the published protocol^(38)^ and outcome papers^(39,40)^ and are summarised below (See ‘Intervention trial outcomes’)

**Figure 1.**
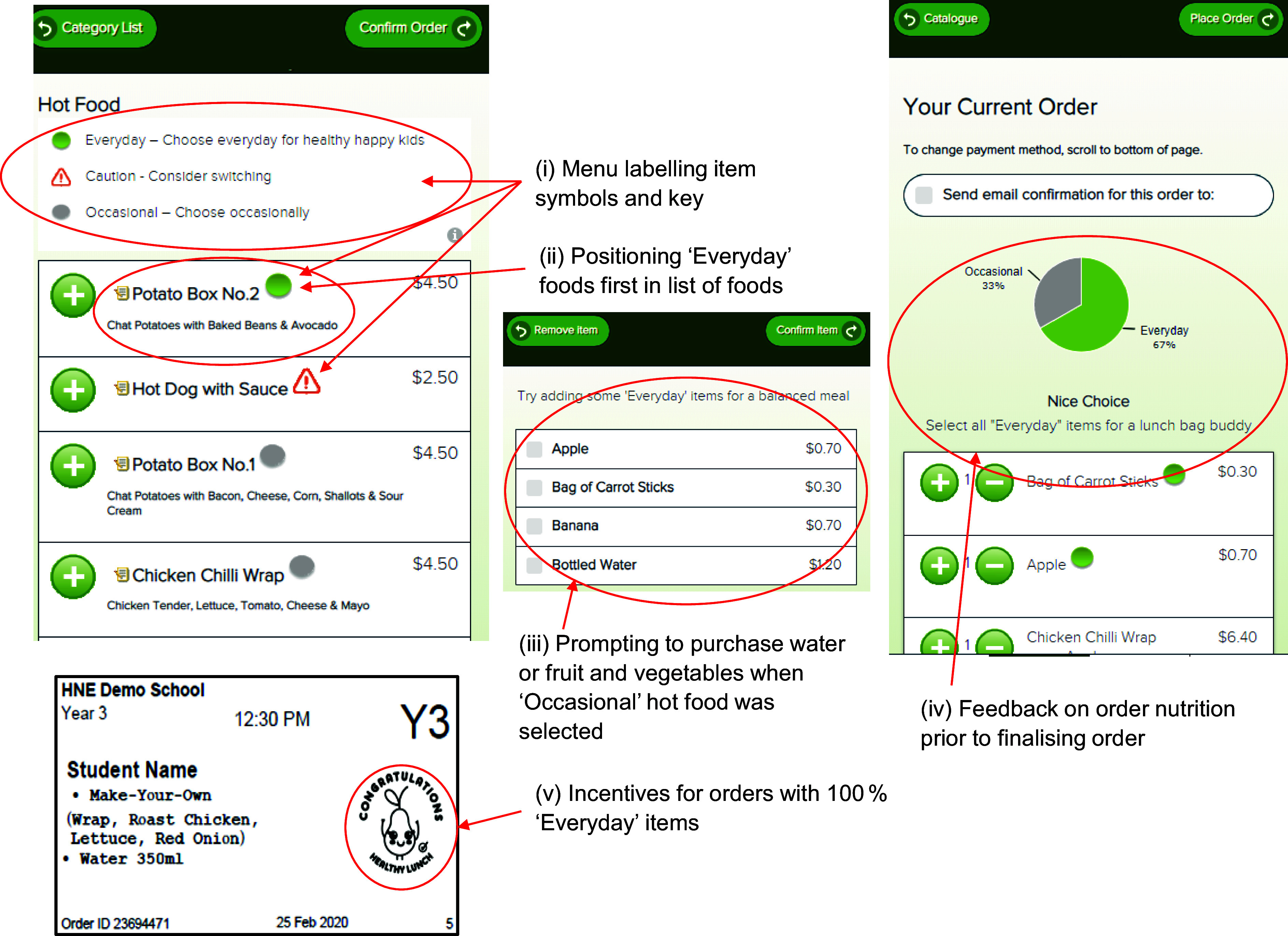
Examples of strategies applied to online menus in the ‘Click & Crunch’ online canteen intervention.

Schools randomly assigned to receive the ‘Click & Crunch’ intervention had modifications made to the layout and presentation of items on their online menu by the online canteen provider to encourage users to select healthier menu items. The choice architecture modifications included menu labelling, item positioning, prompting, feedback and incentives (see Table 1 and Figure 1). Furthermore, canteen managers also received a copy of their labelled canteen menu, a ‘menu audit and feedback report’ and a phone call to discuss the ‘Click & Crunch’ strategies prior to the modifications being made to the online menu. Intervention strategies were based on the menu classification system of the NSW Healthy School Canteen Strategy (3rd Edition)^(41)^ which classifies healthy menu items as ‘Everyday’ and less healthy items as ‘Occasional’. Items which did not meet the criteria within these categories were classified as ‘Should not be sold’.

**Table 1.** Overview of the ‘Click & Crunch’ online canteen intervention

Underpinning theory	• Behavioural Economics, Choice Architecture
Underpinning policy	NSW Healthy School Canteen Strategy: ‘Everyday’ foods form at least 75 % of the canteen menu ‘Should not be sold’ items are removed from menus
Intervention length	8–10·5 months (3–4 school terms from mid-2018 to mid-2019)
*Strategies*	
Menu labelling	A coloured symbol labelling an item as ‘Everyday’, ‘Occasional’ or ‘Should not be sold’ (based on the NSW Healthy Canteen Strategy) was added next to every item on the online menu, along with a key to explain each symbol. ‘Should Not Be Sold’ items were referred to as ‘Caution’ within the online canteen system.
Positioning	Menu items (e.g. ‘Ham and salad sandwich’) and categories (e.g. ‘Sandwiches and wraps’) were arranged to make healthier ‘Everyday’ options more prominent (i.e. positioned first within the list of menu items)
Prompting	An appealing image and brief text prompt were placed next to all healthy categories Users were prompted to purchase water and/or a piece of fruit or vegetables (‘Healthy add-ons’) when they selected an ‘Occasional’ or ‘Should not be sold’ hot food item Prompts were manually implemented on each school’s menu individually
Feedback	Prior to finalising the lunch order, the user was shown a pie graph and tailored feedback based on the proportion of ‘Everyday’ items in the order
Incentives	Orders that contained 100 % ‘Everyday’ items had a cartoon character and congratulatory text printed on the lunch label, which was printed out and stuck on the paper bag in which the lunch order was delivered to students
Supportive strategies	A menu audit and feedback report was sent to canteen managers and principals classifying each item on the menu as per the NSW Healthy School Canteen Strategy (i.e. ‘Everyday’, ‘Occasional’, ‘Should not be sold’) The report also provided general information about pricing items to encourage healthy purchases

NSW, New South Wales.

Schools randomly assigned to the control condition did not have any changes made to their online canteen menu and did not receive any support strategies (e.g. no menu audits or feedback reports).

#### Measurement and analysis of trial outcomes

The effectiveness of the ‘Click & Crunch’ intervention was evaluated using a cluster randomised controlled trial, by analysing objective purchasing data that was automatically collected and stored by the online canteen ordering system. Data were collected simultaneously for all schools, for two 8-week periods, 12 months apart (term 2, 2018, and term 2, 2019). Baseline data were retrospectively collected. Baseline data (2018) included orders for students in Kindergarten to Grade 5 only, as students in Grade 6 would no longer be in primary school at the follow-up data collection period

Primary outcomes for the trial included the mean energy (kJ), saturated fat (g), sugar (g) and sodium (mg) of student online lunch orders. Secondary outcomes were the proportion of items ordered by students that were classified as ‘Everyday’, ‘Occasional’ and ‘Should not be sold’ according to the NSW Healthy Canteen Strategy^(41)^, and the mean proportion of energy within lunch orders derived from saturated fat (calculated as 37 kJ/g) and sugar (calculated as 17 kJ/g)^(42)^

Full details of the data collection procedure, measures, statistical analyses and results have been previously published^(39)^. In summary, an intention-to-treat approach was used for all analyses. Separate linear mixed models were used for the four primary trial outcomes (mean energy, saturated fat, sugar, sodium per student lunch order) and two secondary outcomes (mean proportion of energy from saturated fat and mean proportion of energy from sugar) to compare orders placed by the intervention and control groups throughout time. Three separate logistic regressions were used to assess whether there was a significant change in the proportion of ‘Everyday’, ‘Occasional’ and ‘Should not be sold’ items purchased between groups and included a group-by-time interaction fixed effect. A per-protocol analysis was also conducted to determine the effect of the intervention on two outcomes (mean energy (kJ) of orders and proportion of ‘Everyday’ items purchased) when the intervention was applied in full (i.e. all strategies applied)

### Economic study

A prospective trial-based economic evaluation of the ‘Click & Crunch’ online canteen intervention *v.* usual online canteen practice was conducted from both health service and societal perspectives. This included costs incurred by the health service and the opportunity costs for the school for any time taken to implement the intervention. Intervention development costs and research costs associated with the intervention evaluation were excluded, as were one-off programming costs incurred by the online canteen provider as these costs would not be borne by future schools looking to implement the intervention. The time horizon for the study was 16 months, which includes the two 8-week periods, 12 months apart.

The outcomes of the economic analysis were the direct costs associated with implementing the intervention and incremental cost-effectiveness ratios (ICER) calculated as the ‘difference of the mean total cost’ divided by the observed difference in the (1) mean energy (kJ) content per lunch order and (2) proportion of healthier ‘Everyday’ items purchased. Cost and cost-effectiveness outcomes were calculated at the analysis level of the student. The cost per student was calculated as the cost per school divided by the number of economics students. All outcomes were reported as at 12-months post-baseline

#### Intervention implementation costs

Implementation costs were divided into three categories: (1) collecting menu information; (2) assessing menus; and (3) executing the intervention strategies. Costs incurred by both the health service and canteen managers/schools within these three categories can be seen in Table 2. In this study, a health service project officer was responsible for applying intervention strategies to menus in the online canteen system; therefore, no implementation costs were incurred by the online canteen provider. Data regarding the time taken for each implementation cost category were obtained from project records and valued using award wage rates. The majority of costs were recorded at the time at which they were incurred and then transferred to an ‘Economic Evaluation and Project Management Cost Capture Template’ at the conclusion of the trial.

**Table 2.** Assumptions and sources of unit costs

Intervention category	Details of cost	Source	Unit costs	Cost incurred by
Collecting menu information	Project staff time to collect menus, schedule calls to canteen managers and generate template of menu information to be collected	Project records	HEW 5/Step 1 Fixed Term (+30 % oncosts) $47 per hour HEW 5/Step 1 Casual (+16 % oncosts) $53 per hour	Health service
	Cost of phone calls to collect menu information	Project records	Local telephone call costs $0·30 per call	Health service
	Opportunity cost of canteen managers’ time associated with providing menu information	Estimates from project records	Fast Food Industry Award – Level 2 (+30 % oncosts) $29 per hour	Canteen managers/schools
Assessing menus	Project staff time taken to classify menu items against the NSW canteen guidelines	Project records	HEW 5/Step 1 Casual (+16 % oncosts) $53 per hour	Health service
Executing the intervention strategies	Project staff time to schedule feedback report calls, explain intervention group allocation, generate a digital summary of item classifications, generate a feedback report and discuss report with canteen manager. Switch on intervention strategies in online canteen software	Project records	HEW 5/Step 1 Fixed Term (+30 % oncosts) $47 per hour HEW 5/Step 1 Casual (+16 % oncosts) $53 per hour HEW 7/Step 1 Fixed Term (+30 % oncosts) $60 per hour*	Health service
	Cost of phone calls to discuss feedback reports	Project records	Local telephone call costs $0·30 per call	Health service
	Opportunity cost of canteen manager time associated with discussing the feedback report and intervention	Estimates from project records	Fast Food Industry Award – Level 2 (+30 % oncosts) $29 per hour	Canteen managers/schools

HEW, Health Education Worker; NSW, New South Wales.

Resource-associated costs included project staff salaries, phone calls and canteen manager opportunity costs. The time taken to execute each intervention component was extracted from project records including school contact logs and intervention monitoring spreadsheets maintained by the project staff. Where time data were not recorded (e.g. time taken to assess one school’s menu), estimates were informed by the time recorded for the equivalent steps for another school in the trial with similar characteristics (e.g. a similar number of menu items). The time taken was multiplied by the relevant hourly staff rates including oncosts. Unit costs for project staff were based on the 2018 Higher Education Worker pay scale for fixed term and casual staff. Unit costs for canteen managers were based on Level 2 of the 2018 Fast Food Industry award wage^(43)^. All costs were reported in 2018 Australian Dollars (AUD$). The total cost to implement the intervention was calculated overall, per school and per student. It was assumed that no usual practice costs to schools were displaced as a result of the intervention. Participation in ‘Click & Crunch’ did not come at a cost for the schools.

#### Cost-effectiveness analysis

Economic analyses were undertaken using Microsoft Excel software 2013. The analyses included calculating the total direct health sector costs and total societal costs (i.e. health service plus school costs) of implementing the intervention. The total cost of the intervention was calculated for all students in the intervention group who made at least one online canteen purchase at baseline.

The incremental cost of the intervention was calculated as the difference between the mean cost per student in the intervention group and the mean cost per student in the control group. The incremental health effect was calculated as the difference between the intervention and control groups for the two study outcomes: (1) mean energy (kJ) content of student lunch orders and (2) the mean proportion of healthier ‘Everyday’ items. The ICER represented the difference between the mean cost per student per group divided by the difference between the health effect per group (Intervention cost – control cost/Intervention effect – control effect). If the incremental cost of the intervention is negative (i.e. it costs less) and the incremental health effect of the intervention is positive (i.e. it is more effective), the intervention is considered cost-effective. Alternatively, if the incremental cost is positive (i.e. it costs more) and the incremental health effect is positive (i.e. it is more effective), the intervention can still be considered cost-effective provided the additional cost is below the threshold that society is willing to pay for the additional unit of health outcome (see cost-effectiveness acceptability curves below)^(44)^.

#### Uncertainty analyses

To account for uncertainty that could be due to sampling variation, non-parametric bootstrapping analysis with > 1000 iterations was used to generate uncertainty intervals (UI) around the calculated mean costs and ICER, as well as a scatterplot of incremental cost and incremental health effects. Based on the bootstrapped ICER, a cost-effectiveness acceptability curve was generated which indicated the probability of the intervention being cost-effective at various levels of society’s willingness to pay for (1) a unit reduction in energy (kJ) content per lunch order and (2) a unit increase in the proportion of ‘Everyday’ items purchased.

#### Sensitivity analyses

Sensitivity analyses were conducted to provide an indication of the factors potentially influencing the cost-effectiveness analysis results. Several one-way sensitivity analyses were undertaken to account for higher canteen manager wages and scenarios where menu assessment was not required as part of the intervention. For example, the scenario where schools already had ‘Everyday’/’Occasional’/’Should not be sold’ labelling applied to their menus, as all NSW schools have access to a free ‘Menu Check’ service which classifies their canteen menu based on the NSW Strategy^(45)^. A per-protocol analysis was also conducted to determine the ICER based on the five intervention schools where all intervention strategies were applied and maintained for the duration of the trial.

##### Missing data

A last observation carried forward method was used to account for missing data at follow-up, by imputing the baseline values for energy (kJ) and proportion of ‘Everyday’ items purchased. The last observation carried forward method was considered suitable for this context as it conservatively estimates no change in average dietary intake for a student.

## Results

### Schools and participants

In summary, seventeen non-government primary schools (nine intervention schools and eight control schools) were recruited to the study. In total, 2207 students from Kindergarten to Grade 5 placed at least one online lunch order during the 8-week baseline data collection period (1359 intervention school students, 848 control school students) and 82 % ordered within the follow-up period. Seven schools (three intervention schools, four control schools) were located in areas of less socio-economic advantage according to the Australian Socio-Economic Indexes for Areas^(46)^. There was a paid canteen manager (as opposed to an unpaid for, for example, a parent or carer from within the school community) in all schools within the trial, and the canteen was open 5 d a week (all school days) in eleven of the participating schools.

### Intervention trial outcomes

Over time, the between-group difference in energy content of online lunch orders was –69 kJ (95 % CI: –120, –19, *P* = 0·01), and the odds of purchasing ‘Everyday’ items was greater in intervention schools relative to control schools (OR = 1·7, 95 % CI: 1·5, 2·0, *P* < 0·001) which corresponded to a 9·8 % increase in ‘Everyday’ item purchases. A per-protocol analysis was conducted incorporating five intervention schools, and this increased the between-group difference in lunch order energy content to –89 kJ (95 % CI: –149, –30, *P* = 0·007). The odds of purchasing ‘Everyday’ items remained similar (OR = 1·5, 95 % CI: 1·3, 1·8, *P* < 0·001).

### Economic study outcomes

#### Intervention costs

The total costs (by category) of implementing the ‘Click & Crunch’ online canteen intervention can be seen in Table 3. The total overall cost to implement the intervention was calculated to be $5111, which equated to an average of $568 per school (range $343–$806). Of the $568 cost, $518 was attributable to the health service and $50 attributable to the canteen manager/school. The average cost of switching on the intervention strategies in the online canteen system was $112 per school and was incurred by the health service (i.e. the time taken to make the changes to each school’s online menu).

**Table 3.** Total costs of delivering the Click & Crunch intervention

	Incurred by	Wages total cost (AUD$)	Phone calls total cost (AUD$)	Overall total cost (AUD$)	Overall cost per school (*n* 9) (AUD$)
Collecting menu information	Health service	981	16	997	111
	Canteen managers & schools	199	–	199	22
Assessing menus	Health service	1202	–	1202	134
Executing the intervention	Health service	2455	11	2466	274
	Canteen managers & schools	247	–	247	27
Total cost incurred by health service		4638	27	4665	518
Total cost incurred by canteen managers & schools		446	–	446	50
Total cost of intervention		5084	27	5111	568

#### Incremental Cost-Effectiveness Ratios

From a health service perspective, the incremental cost per kJ reduction in lunch orders associated with the intervention was $0·06 per student (UI: $0·05–$0·09), and the incremental cost per increase in the proportion of ‘Everyday’ items purchased was $0·46 per student (UI: $0·41–$0·52). From a societal perspective, the incremental cost per kJ reduction in lunch orders associated with the intervention was $0·07 per student (UI: $0·06–$0·10), and incremental cost per unit increase in the proportion of ‘Everyday’ items purchased was $0·50 per student (UI: $0·45–$0·57).

**Figure 2.**
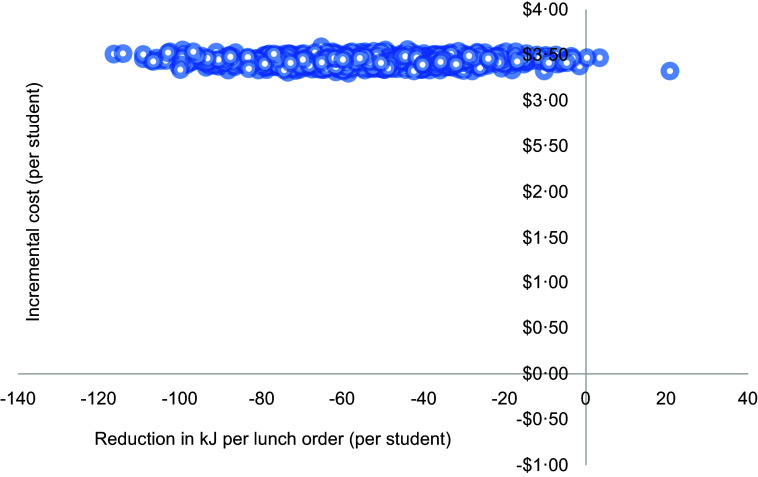
Cost-effectiveness plane for reduction in kJ per lunch order.

**Figure 3.**
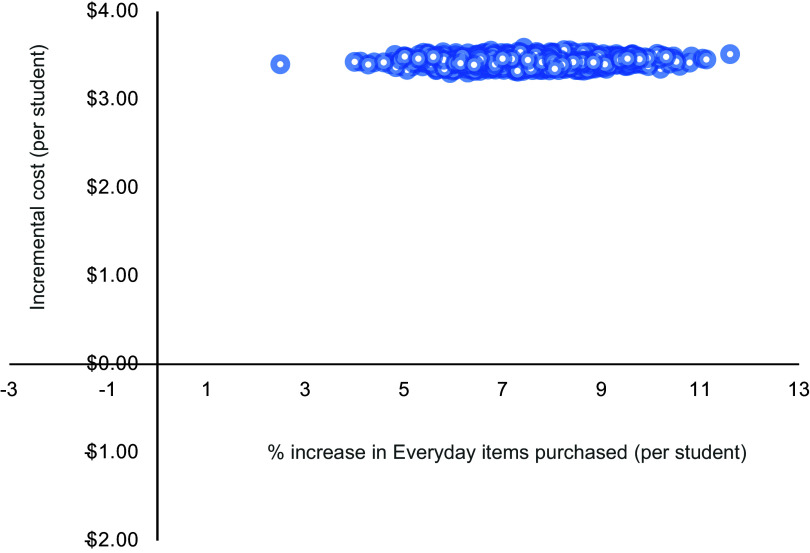
Cost-effectiveness plane for an increase in the proportion of ‘Everyday’ items purchased.

The cost-effectiveness planes (Figures 2 and 3) showed the intervention was both more costly and more effective than usual online canteen ordering for both primary outcomes (reduction of average energy content per lunch order and increase in the average proportion of ‘Everyday’ items purchased).

**Figure 4.**
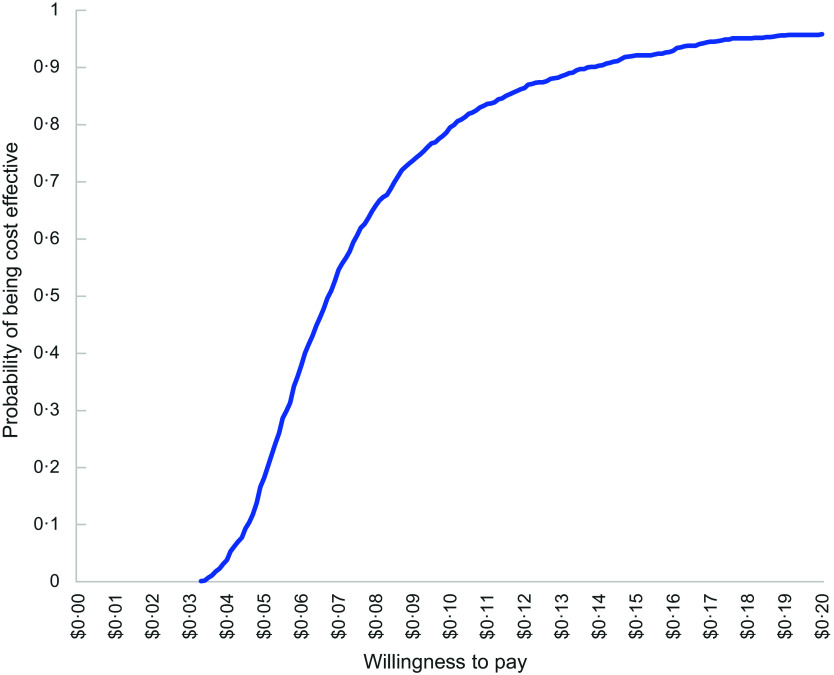
Cost-effectiveness acceptability curve per reduction in energy (kJ).

**Figure 5.**
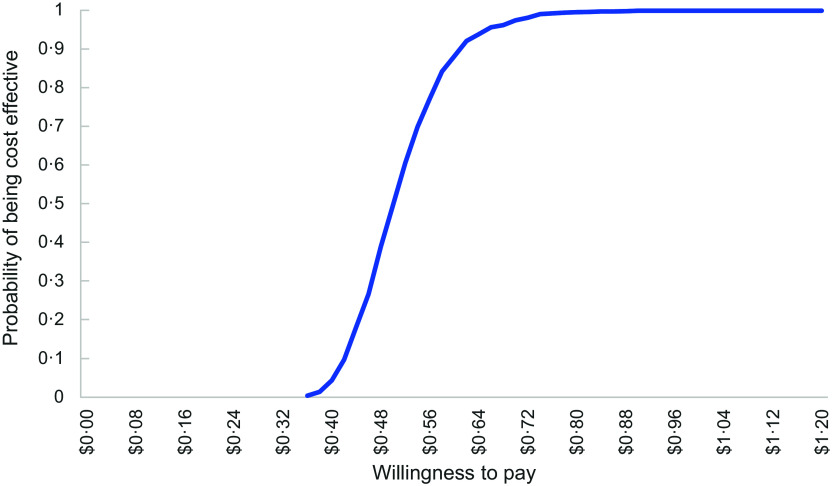
Cost-effectiveness acceptability curve per increase in ‘Everyday’ items purchased.

The cost-effectiveness acceptability curves (Figures 4 and 5) indicate the likelihood that the intervention would be considered cost-effective across a range of costs per student (AUD) for the primary outcomes. Per unit reduction in energy (kJ) per lunch order, the intervention would have a 95 % probability of being cost-effective at a cost of AUD $0·20 per student. Per percentage increase in the proportion of ‘Everyday’ items purchased, the intervention would have a 99 % probability of being cost-effective at a cost of AUD $1·20 per student.

#### Sensitivity analyses

When sensitivity analyses were conducted to determine the impact of higher canteen manager salaries on societal costs, the incremental cost per unit reduction in kJ per order remained stable at $0·07 per student (UI: $0·06–$0·09), as did the incremental cost per percentage increase in the proportion of ‘Everyday’ items purchased at $0·50 per student ($0·45–$0·56), from a societal perspective.

The removal of project personnel costs related to the menu assessment component of the intervention (i.e. to reflect the scenario where menu assessment was not required as part of the intervention) resulted in a $0·01 per student lower calculated direct health service cost per unit reduction in kJ ($0·05, UI: $0·04–$0·06) and a $0·12 per student lower estimated direct health service cost per percentage increase in the proportion of ‘Everyday’ items purchased ($0·34, UI: $0·30–$0·38). From a societal perspective, there was a $0·02 per student reduction in costs per unit reduction kJ per order ($0·05, UI: $0·04–$0·07) and a $0·12 per student reduction in costs per increase in the proportion of ‘Everyday’ items purchased ($0·38, UI: $0·34–$0·43).

A per-protocol approach to the analyses resulted in a $0·01 per student lower calculated direct health service cost per reduction in kJ per order ($0·05, UI: $0·04–$0·07) and a $0·09 per student lower calculated direct health service cost per increase in the proportion of ‘Everyday’ items purchased ($0·37, UI: $0·32–$0·41). From a societal perspective, there was a $0·01 per student reduction in costs per reduction in kJ per order ($0·06, UI: $0·05–$0·08) and a $0·10 reduction in cost per increase in the proportion of ‘Everyday’ items purchased ($0·40, UI: $0·36–$0·45).

## Discussion

### Principle results

This study aimed to determine the cost and cost-effectiveness of implementing an online canteen ordering intervention to improve the nutritional quality of lunch orders purchased for primary-school-aged children. To the best of the authors’ knowledge, this is the first time such analysis has been conducted on an intervention delivered through an online food ordering system in schools. The mean total cost per school to implement the intervention was AUD$568 (range $343–$806). The cost per student to achieve a 1 kJ reduction in the average energy content of their online lunch order was calculated to be AUD$0·06 from a health perspective and AUD$0·07 from a societal perspective. The cost per student, to achieve a 1 % increase in the average proportion of ‘Everyday’ items purchased in their online lunch orders, was calculated to be AUD$0·46 and AUD$0·50 from a health perspective and societal perspective, respectively. Per-protocol and sensitivity analyses had only minimal impact on the ICER for a reduction in average energy content, but a larger reduction in the ICER for the proportion of ‘Everyday’ items purchased, with the range of decrease between AUD$0·10–$0·12.

### Comparison with prior work

This study is the first economic evaluation of a public health intervention delivered to primary school students using online canteen ordering systems. This is important given the proliferation of online food ordering systems within schools and the increasing number of users and the increasing volume of food purchased using these systems each year, and given evidence that public health nutrition interventions delivered within these systems are effective (as per meta-analysis, Wyse *et al.*, 2021^(11)^) and other trials in the setting^(47,48)^.

Economic analyses have previously been conducted for non-online canteen interventions in NSW primary schools. Reilly *et al.* examined the cost of three interventions of differing intensity that aimed to increase school implementation of a state-wide food availability policy. The average cost per school to deliver each type of intervention was calculated at: AUD$4771 (high intensity); AUD$2216 (medium intensity); and AUD$2102 (low intensity), compared to the lower cost to implement the ‘Click & Crunch’ intervention of AUD$568 per school. Reilly et al also calculated the average cost-effectiveness ratio for each type of intervention to achieve a 1 % percent increase in the proportion of schools adherent to the policy: AUD$2982 (high intensity), AUD$2627 (medium intensity) and AUD$4730 (low intensity). However, the unit of analysis of the Reilly et al trials (i.e. the proportion of policy-adherent schools) and its canteen manager-focused intervention (*v*. the consumer-focused approach) make it difficult to compare to the average cost per school and the ICER of the ‘Click & Crunch’ intervention.

Another mobile health intervention in NSW (SWAP IT) aimed to improve the nutritional quality of packed school lunchboxes (i.e. foods brought from home, rather than purchased from a canteen or school food outlet) and achieved a 57 kJ reduction in average energy content in intervention lunchboxes. The accompanying economic analysis found that the incremental cost per student to achieve a 1 kJ reduction in energy content was AUD$0·54 (Brown *et al* ^(49)^). This represents a higher incremental cost per student than the online canteen intervention at AUD$0·06 per kJ unit reduction. The Cost-Effectiveness Acceptability Curve for the SWAP IT indicated that assuming a willingness to pay of AUD$40 per person, the lunchbox intervention would have a 99 % probability of being cost-effective. This compares to a willingness to pay of AUD$0·20 per student having a 95 % probability of being cost-effective for the ‘Click & Crunch’ online canteens intervention. However, these differences should be considered with respect to the frequency of online canteen use; 34 % of users order from the online canteen once or more a week, compared to the more frequent use of lunchboxes brought from home. We were unable to find any published economic evaluations of online school food service interventions which included a cost-effectiveness acceptability curve, against which to compare our results.

### Implications

This study highlights some additional considerations for any future roll-out of this or similar interventions. The detailed breakdown of the costs to deliver the ‘Click & Crunch’ intervention indicates most of the costs incurred were due to canteen menu collection and dietitian assessment of the menu (i.e. collecting menus, scheduling calls to canteen managers, generating an individual template of menu information to be collected per school and then assessing each menu against NSW canteen guidelines). This research trial required the precise calculation of the nutritional composition of all canteen menu items (i.e. kJ, saturated fat, sodium and sugar content). However, this calculation process was more detailed and therefore more time-consuming than other forms of menu assessment currently employed by the state health service. For example, at the time of this trial, the NSW Government provided a free state-wide ‘Menu Check Service’, which classified each item on a school’s canteen menu as ‘Everyday’, ‘Occasional’ and ‘Should Not Be Sold’. Since 2021, over 94 % of government schools have utilised the Menu Check Service^(50)^. The simpler menu assessment provided by the ‘Menu Check Service’ would be sufficient to implement the ‘Click & Crunch strategies’. However, in order to evaluate the primary outcomes of the research trial, a more detailed quantitative summary of the nutrient composition of all menu items was necessary, which was very time-consuming. The sensitivity analysis indicated that dropping this stringent menu assessment would result in a $0·01 decrease in the ICER for energy content, representing a 17 % increase in cost-effectiveness. Furthermore, the majority of execution costs were attributable to the implementation of strategies that had to be entered manually (i.e. prompts that were tailored to specific items on each school’s menu). Further research should be conducted to determine the incremental effectiveness of any manually entered strategies. In this study, project officer time, representing a cost to the health service, was used to apply the intervention strategies to the online ordering system at an average cost of $112, assuming salary costs of $60/h. These data may be useful for stakeholders to design future implementation models and explore alternative models where the implementation costs are borne by either the schools, the health service or the online providers.

### Strengths and limitations

The findings of this study should be considered with respect to the strengths and limitations of the design and execution. Although a large number of students participated in the trial, the number of participating schools was small and there was substantial variation in the costs incurred between schools (range AUD$343–$806). This was largely due to variations in the number of items available on the menus at different schools. Furthermore, although schools from a variation of geographic regions participated, the trial only included non-government schools, and it is unclear whether these results would also apply to government schools. The calculated costs did not include intervention development or research costs because these would not be incurred when the intervention was implemented at schools in the future. Furthermore, any adjustments to food preparation routines that canteen managers made as a result of the intervention were not assessed. For example, there were anecdotal reports that participation in the intervention resulted in some changes in food preparation for some canteen managers. For example, in intervention schools, two production lists were generated rather than the standard one. Receiving ‘Click & Crunch’ may have caused or influenced broader changes within intervention schools that we were unable to capture. For example, intervention strategies may have caused canteen managers to source new products or suppliers which may have affected their costs. It is important to note that the societal perspective analysis did not include the time taken by system users (i.e. parents and students) to place an online lunch order. However, as the intervention is based on choice architecture strategies, and cognitive processing of these strategies is quick and largely automatic, this was considered to be negligible. Also, while clustering was accounted for in the univariate analysis of outcomes, the same statistical treatment was not applied to the analysis of cost^(51)^. While non-parametric bootstrapping was employed to represent uncertainty, it is possible that this method underestimated the uncertainty, as shown by Gomes et al^(51)^. Finally, the analysis includes only those food and beverage items that were purchased via the online ordering system. Schools may still allow over-the-counter purchases at lunchtime, and as such, these would not have been included. A number of areas for additional research were identified. The uptake rate of online canteen ordering among very disadvantaged schools is unknown, and further research should investigate this to determine the equity of this intervention approach. Furthermore, the change in the intervention effect and therefore the cost-effectiveness of the intervention over time are not known. This study assessed changes in purchasing patterns in corresponding school terms, one year apart. However, seasonality and the effectiveness of the intervention may change based on time and time of year and should be investigated further.

The strengths of this economic analysis include the use of a cluster randomised controlled trial design with a large number of students and student lunch orders collected from schools across a diversity of regions. Furthermore, a detailed cost capture template was used to record costs, with the majority of information recorded at the time the costs were incurred. This analysis also includes sensitivity analysis to account for the varying costs of inputs and a per-protocol analysis to account for variation in levels of intervention fidelity, and the analysis also considers both a health and societal perspective.

### Conclusions

This economic analysis provides the first evidence of the cost and cost-effectiveness of implementing a public health nutrition intervention via a school canteen online ordering system. The ‘Click & Crunch’ intervention was more costly but more effective than the standard online canteen ordering system, and assuming a willingness to pay of AUD$0·20 per student per kJ unit reduction and AUD$1·20 per student per percentage increase in the average proportion of ‘Everyday’ items, the intervention would have a greater than 95 % and 99 % probability, respectively, of being cost-effective. Findings from this study highlight the potential of online food ordering systems for delivering public health nutrition interventions. Further research into the effectiveness, cost-effectiveness and willingness to pay for such interventions is warranted.
